# Cognition and violent behavior in psychotic disorders: A nationwide case-control study

**DOI:** 10.1016/j.scog.2019.100166

**Published:** 2019-10-31

**Authors:** Jelle Lamsma, Wiepke Cahn, Seena Fazel, Genetic Risk and Outcome of Psychosis (GROUP) investigators

**Affiliations:** a Department of Psychiatry, University of Oxford, Oxford, UK; b Department of Psychiatry, University Medical Center Utrecht, Utrecht, the Netherlands

**Keywords:** Schizophrenia, Psychosis, Executive functions, Theory of mind, Aggression, Violence

## Abstract

**Background:**

The excess risk of violence in psychotic disorders may partly be explained by impairments in executive functions (EFs) and theory of mind (ToM). However, previous studies have been limited by composite measures of EFs and small samples of inpatients.

**Methods:**

Data were collected for the research project Genetic Risk and Outcome of Psychosis (GROUP). Patients with psychotic disorders (*N* = 891) were recruited from various care settings in the Netherlands. The following neuropsychological tests were administered (targeted cognitive function in parentheses): (i) Continuous Performance Test-HQ (inhibition); (ii) Response Shifting Task (cognitive flexibility); (iii) Wechsler Adult Intelligence Scale, Third Edition (WAIS-III) Block Design subtest (fluid intelligence); (iv) Neuropsychological Assessment Battery (NAB) Mazes Test (planning); (v) Degraded Facial Affect Recognition Task (affective ToM); and (vi) Hinting Task (cognitive ToM). Lifetime violence was ascertained from medical records and patient interviews. We used analysis of covariance to compare the mean scores of violent and nonviolent patients on each test, adjusting for age and sex.

**Results:**

Violent patients performed significantly worse than nonviolent patients on the WAIS-III Block Design subtest (*F* [1, 847] = 5.12, *p* = .024), NAB Mazes Test (*F* [1, 499] = 5.32, *p* = .022) and Hinting Task (*F* [1, 839] = 9.38, *p* = .002). For the other tests, the between-group differences were nonsignificant. Violent behavior explained no more than 1% of the variance in performance on each test.

**Conclusion:**

Impairments in EFs and ToM are unlikely to provide useful targets for risk assessment and interventions.

## 1 Introduction

People with psychotic disorders are at increased risk of violent behavior compared with the general population (Fazel et al., 2009; Large and Nielssen, 2011). In a meta-analysis of 20 studies with a total of 18 423 cases and 1 714 904 unaffected controls, the odds ratio for violence in schizophrenia was 5.5 (95% confidence interval [CI] 4.1–7.5) and in other psychotic disorders 4.9 (95% CI 3.6–6.6)(Fazel et al., 2009). More recent studies have confirmed this risk using family-based designs (Fazel et al., 2014a; Sariaslan et al., 2016). Several risk factors for violence in psychotic disorders have been identified, including previous crime, symptoms of delusions and hallucinations, lack of insight and substance misuse (Witt et al., 2013). Potentially important, but rarely studied, are impairments in executive functions (EFs) and theory of mind (ToM).

EFs are mental operations needed to direct behavior toward the realization of goals (Miller and Wallis, 2009). There are three elementary EFs: inhibition, working memory and cognitive flexibility. These combine to build the higher order EFs of fluid intelligence (i.e. reasoning and problem solving) and planning (Diamond, 2013). ToM is the ability to infer mental states (e.g. motivations, emotions) in oneself and others (Fonagy and Allison, 2012). Two types of ToM can be distinguished, according to whether the mental state being inferred is affective (i.e. affective ToM) or cognitive (i.e. cognitive ToM) in nature (Shamay-Tsoory et al., 2010).

Since EFs and ToM are essential for social adaptation, impairments in these cognitive functions are thought to underlie violent behavior. However, causal mechanisms likely differ by EF (Raine, 2008) and ToM type (Adshead et al., 2013). General population studies have repeatedly found that people who display violent behavior perform worse on neuropsychological tests of EFs (Ogilvie et al., 2011) and ToM (Hoaken et al., 2007; Nyline et al., 2018) than those who do not. A meta-analysis of 126 studies totaling 14 786 participants reported a significant inverse association between test performance and antisocial behavior (including violence) across EFs. This association had a medium effect size (*d* = 0.4, 95% CI 0.4–0.5) (Ogilvie et al., 2011).

Impairments in EFs and ToM are a core feature of psychotic disorders (Kahn and Keefe, 2013). An umbrella review of 10 meta-analyses found that individuals with schizophrenia performed between 0.5 and 1.5 standard deviations below unaffected controls on tests of EFs (Reichenberg and Harvey, 2007). Meta-analyses of ToM have reported similar effect sizes (Chung et al., 2014; Sprong et al., 2007). By and large, EF and ToM deficits are present before illness onset (Kahn and Keefe, 2013), stable over time (Reichenberg, 2011) and independent of psychotic symptoms (Nieuwenstein et al., 2001) and treatment with antipsychotic medication (Mishara and Goldberg, 2004). Furthermore, they are associated with adverse outcomes such as rehospitalization (Harvey et al., 2013), substance misuse (Eack et al., 2015) and low educational attainment (Keefe, 2008).

Based on this, it may be hypothesized that EF and ToM impairments partly explain the excess risk of violent behavior in psychotic disorders. The few studies investigating this hypothesis have produced mixed results for EFs (Barkataki et al., 2005; Fullam and Dolan, 2008; Lapierre et al., 1995; Krakowski et al., 2016; Serper et al., 2008) and affective ToM (Abu-Akel and Abushua'leh, 2004; Frommann et al., 2013; Silver et al., 2005; Weiss et al., 2006). However, these studies were limited by small samples of male inpatients or prisoners, a single data source of violent behavior and composite measures of EFs. To our knowledge, there has only been one study of cognitive ToM. This study reported a significant association between higher scores on the Unexpected Transfer Task (Perner and Wimmer, 1985) and lifetime violence (relative risk = 1.2, *p* < .05), ascertained from medical records and interviews with hospital staff, in patients with schizophrenia. This finding may be attributed to selection bias: the sample size was small (*N* = 24), and the violent patients were recruited from a high-security psychiatric hospital (Abu-Akel and Abushua'leh, 2004).

To address the limitations of previous studies, we have investigated the association between neuropsychological test performance and a sensitive marker of violent behavior for a comprehensive set of EFs and affective and cognitive ToM in a large nationwide sample of people with psychotic disorders.

## 2 Methods

### 2.1 Setting and participants

Data (release 5.0) were collected as part of a larger project, called Genetic Risk and Outcome in Psychosis (GROUP). GROUP is conducted by the psychiatry departments of 4 university medical centers (i.e. Amsterdam Medical Center, Maastricht University Medical Center+, University Medical Center Groningen, University Medical Center Utrecht) and their affiliated mental health centers (*k* = 36) in the Netherlands. Together, these centers provide access to inpatient or outpatient treatment to approximately 75% of the Dutch population. Assessments took place at baseline (2004) and after three and six years of follow-up. Eligible patients were identified by screening clinicians' caseloads for the following criteria: (i) age between 16 and 50; (ii) good command of the Dutch language; and (iii) Diagnostic and Statistical Manual of Mental Disorders, Fourth Edition, Text Revision (DSM-IV-TR) (American Psychiatric Association, 2000) diagnosis of schizophrenia or another psychotic disorder. There were no exclusion criteria. The protocol was approved centrally by the ethics committee of the University Medical Center Utrecht, and all patients gave written informed consent before the first assessment.

### 2.2 Measures

The psychometric properties of the instruments and training of research personnel have been described elsewhere (Korver et al., 2012).

#### 2.2.1 Neuropsychological tests

We chose tests whose targeted cognitive functions (in parentheses) are hypothetically related to violent behavior: (i) Continuous Performance Test-HQ (CPT-HQ) (Nuechterlein and Dawson, 1984) (inhibition); (ii) Response Shifting Task (RST) (Bilder et al., 1992) (cognitive flexibility); (iii) Wechsler Adult Intelligence Scale, Third Edition (WAIS-III) (Wechsler, 1997) Block Design subtest (fluid intelligence); (iv) Neuropsychological Assessment Battery (NAB) (Stern and White, 2003) Mazes Test (planning); (v) Degraded Facial Affect Recognition Task (DFAR) (van't Wout et al., 2004) (affective ToM); and (vi) Hinting Task (Corcoran et al., 1995) (cognitive ToM). The NAB Mazes Test was only administered at the third wave. Information about the testing procedure can be found in the supplement.

#### 2.2.2 Symptom severity Singh et al., 2011

Symptom severity was measured with the Positive and Negative Syndrome Scale (PANSS) (Kay et al., 1987).

#### 2.2.3 Substance misuse

The Substance Abuse Module of the Composite International Diagnostic Interview (World Health Organization, 1990) was used to establish a lifetime history of substance misuse. We defined alcohol misuse as an average intake of more than 18 standard drinks per week for men and more than 12 standard drinks per week for women during a minimum period of 2 weeks in the past year or 4 weeks at any other point in the past. These cutoffs reflect the median of several national guidelines and a consistent 1.5:1 male to female consumption ratio (Furtwaengler and de Visser, 2013). For other substances, misuse referred to a DSM-IV-TR diagnosis of abuse or dependence.

#### 2.2.4 Violent behavior

Violent behavior, defined as the use of physical force with the intention to harm another person, was assessed with the Life Chart Schedule (LCS) (Susser et al., 2000). The reference period was the lifetime. The LCS was filled out based on review of medical records and interviews with the patient and, if possible, one or both parents.

### 2.3 Statistical analysis

In the analyses involving the NAB Mazes Test, we used age, PANSS total score and educational level at the third wave. Otherwise, baseline data were used. Higher scores on all tests reflected better performance, apart from certain subscales of the CPT-HQ (i.e. number of commission errors) and RST (i.e. accuracy cost, reaction time cost). Therefore, these scores were reversed for the current analyses. For the CPT-HQ and RST, we created composite scores by transforming the scores on the subscales to *z*-scores and then averaging the *z*-scores. This method for creating composite scores is widely used (Harrison et al., 2016; Mancuso et al., 2011). To reduce confounding by impairments in face recognition ability, patients with scores below 18 on the Benton Facial Recognition Test (Benton et al., 1983) were excluded from the analyses with the DFAR.

Depending on the scale of measurement, we assessed differences between violent and nonviolent patients on descriptive characteristics with the *χ*^*2*^-test (nominal) or *t*-test (continuous). Analysis of covariance (ANCOVA) was used to compare the mean scores of violent and nonviolent patients on each neuropsychological test. For theoretical reasons, we included age (Henry et al., 2013) and sex (Longenecker et al., 2010) as covariates. We only analysed patients with available data on all model variables. This reduced the total number of patients from 1 013 to 891.

To evaluate the robustness of the results, we repeated the analyses after separately removing: (i) patients whose violence did not result in injury; (ii) patients who had been violent before illness onset; (iii) patients with PANSS total scores of 95 or higher, indicative of “marked or severe illness” (Leucht et al., 2005); (iv) patients who had misused substances; and (v) patients who had not completed secondary education.

All models satisfied the assumptions of ANCOVA (e.g. homoscedasticity, homogeneity of regression slopes). The level of statistical significance was set at 5%. Analyses were carried out in SPSS 21.0.

## 3 Results

Table 1 shows the demographic and clinical characteristics of the patients (*N* = 891) at baseline. The mean age was 27.2 (SD = 7.0). Most patients were male (*n* = 688, 77%) and had received a diagnosis of schizophrenia (*n* = 615, 69%). The prevalence of violent behavior was 21% (*n* = 183). Violent patients were significantly younger (*t* [889] = 2.9, *p* = .004), had higher PANSS total scores (*t* [809] = 3.80, *p* <.001) and were more likely to have misused substances (*χ*^*2*^ [1] = 4.26, *p* = .039) than nonviolent patients.

**Table 1:** Baseline characteristics of patients with psychotic disorders.

	Violent behavior			
	Yes (*n* = 183)	No (*n* = 708)	Test statistic (*df*)	*p*
Demographic characteristics				
Age, mean (SD) in years	25.9 (6.3)	27.6 (7.2)	*t* (889) = 2.9	.004
Male, n (%)	145 (79)	543 (77)	*χ*^*2*^ (1) = 0.53	ns
Caucasian. n (%)	137 (76)	559 (80)	*χ*^*2*^ (8) = 9.66	ns
Completed secondary education, n (%)	146 (80)	620 (88)	*χ*^*2*^ (1) = 8.17	.004
Clinical characteristics				
DSM-IV-TR diagnosis			*χ*^*2*^ (3) = 6.80	ns
Schizophrenia, n (%)	136 (74)	479 (68)		
Schizoaffective disorder, n (%)	18 (10)	90 (13)		
Psychotic disorder NOS, n (%)	18 (10)	57 (8)		
Other, n (%)	11 (6)	82 (12)		
Age of onset, mean (SD) in years	21.7 (5.6)	23.4 (6.6)	*t* (889) = 3.37	.001
PANSS total score (SD)	58.9 (17.1)	53.5 (16.2)	*t* (809) = 3.80	<.001
Substance misuse, n (%)	118 (68)	405 (60)	*χ*^*2*^ (1) = 4.26	.039

*df*, degrees of freedom; SD, standard deviation; ns, nonsignificant; DSM-IV-TR, Diagnostic and Statistical Manual of Mental Disorders, Fourth Edition, Text Revision; NOS, not otherwise specified; PANSS, Positive and Negative Syndrome Scale.

Due to missing data, the total number of patients varies per characteristic.

Violent patients performed worse than nonviolent patients on most neuropsychological tests (Table 2). On average, they had fewer hits and longer reaction times for hits on the CPT-HQ, higher accuracy and reaction time cost scores on the RST and lower scores on the WAIS-III Block Design subtest, NAB Mazes Test and Hinting Task. The mean number of commission errors on the CPT-HQ was lower and the percentage of correctly identified emotions on the DFAR was higher in violent patients, indicating better performance. The between-group differences reached statistical significance for the WAIS-III Block Design subtest (*F* [1, 847] = 5.1, *p* = .024), NAB Mazes Test (*F* [1, 499] = 5.32, *p* = .022) and Hinting Task (*F* [1, 839] = 9.4, *p* = .002). Effect sizes were small: violent behavior explained 1% or less of the variance in performance on each test.

**Table 2:** Neuropsychological test performance in violent and nonviolent patients with psychotic disorders.

		Unadjusted M (SD)		Adjusted M (SE)^a^				
Targeted cognitive function	Neuropsychological test	V (*n* = 183)	NV (*n* = 708)	V (*n* = 183)	NV (*n* = 708)	*F* (*df1*, *df2*)	*p*	η_p_^2^
Executive functions								
Inhibition	CPT-HQ^b^	0.01 (0.52)	0.00 (0.55)	0.02 (0.04)	−0.01 (0.02)	0.33 (1, 782)	ns	<.001
	Number of hits	26.1 (3.0)	26.4 (2.8)					
	Mean reaction time hits^c^	44.0 (8.3)	42.8 (8.7)					
	Number of commission errors	2.6 (10.8)	3.0 (15.3)					
Cognitive flexibility	RST^b^	−0.05 (0.79)	0.01 (0.75)	−0.06 (0.06)	0.02 (0.03)	1.27 (1, 758)	ns	.002
	Accuracy cost	24.4 (25.5)	22.2 (23.6)					
	Reaction time cost^c^	26.2 (18.9)	25.3 (18.9)					
Fluid intelligence	WAIS-III block design subtest	38.2 (17.5)	41.0 (16.9)	37.8 (1.3)	41.1 (0.7)	5.12 (1, 847)	.024	.006
Planning	NAB mazes test^d^	15.9 (6.5)	17.0 (6.3)	15.6 (0.6)	17.1 (0.3)	5.32 (1, 499)	.022	.011
Theory of mind								
Affective	DFAR	69.1 (10.2)	68.9 (10.7)	69.0 (0.8)	68.9 (0.4)	0.01 (1, 761)	ns	<.001
Cognitive	Hinting task	17.0 (2.9)	17.7 (2.7)	17.0 (0.2)	17.7 (0.1)	9.38 (1, 839)	.002	.011

M, mean; SD, standard deviation; SE, standard error; V, violent; NV, nonviolent; *df*, degrees of freedom; CPT-HQ, Continuous Performance Test-HQ; ns, nonsignificant; RST, Response Shifting Task; WAIS-III, Wechsler Adult Intelligence Scale, Third Edition; NAB, Neuropsychological Assessment Battery; DFAR, Degraded Facial Affect Recognition Task.

Due to missing data, the total number of patients varies per test.

a Adjusted for age and sex.

b Average of subscale *z*-scores.

c In centiseconds.

d Administered six years after baseline.

We observed no material differences in results when restricting the analyses to patients whose violence resulted in injury (Table S2), patients who had only been violent after illness onset (Table S3), patients with PANSS total scores below 95 (Table S4), patients without substance misuse (Table S5) or patients who had completed secondary education (Table S6).

## 4 Discussion

In a nationwide sample of 891 patients with psychotic disorders, we have investigated the association between neuropsychological test performance and lifetime violence for a comprehensive range of executive functions and affective and cognitive theory of mind. Violent patients performed significantly worse than nonviolent patients on tests of fluid intelligence (i.e. WAIS-III Block Design subtest), planning (i.e. NAB Mazes Test) and cognitive ToM (i.e. Hinting Task). However, effect sizes were small.

Violent behavior in people with impaired fluid intelligence may be a maladaptive solution to (Weiss, 2012) or consequence of increased stress responsivity in provocative situations (Sandi and Haller, 2015). Planning deficiencies increase the probability of violence by negatively affecting a person's ability to assess the possible consequences of his or her actions (Meijers et al., 2017). Impairments in ToM may lead to violence through misinterpretation of social cues (Adshead et al., 2013), underregulation of negative emotions (Fonagy and Luyten, 2009), blurring of self-other boundaries (Adshead et al., 2013) or lack of empathy (Hooker et al., 2008). There was no significant difference between violent and nonviolent patients in affective ToM, which is arguably more important for the last three than cognitive ToM. This lends weight to misinterpretation of social cues – insofar they relate to other people's cognitive mental states – as a reason for violent behavior in psychotic disorders. Cognitive ToM also subserves insight (Ng et al., 2015) and, relatedly, treatment adherence (Shad et al., 2006). Insight refers to a person's acknowledgement of having an illness that requires treatment (Beck et al., 2004). A person lacking insight may not seek or adhere to treatment, thereby allowing psychotic symptoms to persist or worsen (Higashi et al., 2013). Furthermore, a strong conviction that delusions or hallucinations are real may bring a person to act on them (Bjørkly, 2006). Alternative explanations for the findings are confounding by biological (e.g. genetics, neurobiological abnormalities) or early environmental (e.g. poor nutrition, maltreatment) risk factors (Lamsma and Harte, 2015).

There are several limitations to this study. First, causality cannot be inferred because violent behavior preceded test administration by a potentially long period of time. However, the relative stability of cognition makes this less problematic. Second, the LCS does not distinguish between community and inpatient violence, which may have different cognitive correlates (Weiss, 2012). It has also been suggested that cognitive impairment is more pronounced in patients who persistently display violent behavior from an early age than in those who become violent after illness onset (Hodgins and Klein, 2017). However, we found similar results in the latter. Third, most patients had used antipsychotic medication. This may have attenuated associations, as some antipsychotics – most notably clozapine – have been shown to reduce violence (Bhavsar et al., 2019; Fazel et al., 2014b). However, reported improvements in EFs (Mishara and Goldberg, 2004) and ToM (Kucharska-Pietura and Mortimer, 2013) after treatment with antipsychotics are usually too small to be considered clinically significant. For that reason, we expect attenuation to have been negligible. Fourth, neuropsychological tests have limited construct and ecological validity. The construct validity, or the degree to which an instrument measures what it is designed to measure, of many tests is lowered by systematic variance in performance that is attributable to nontargeted cognitive functions. For example, the WAIS-III Block Design subtest not only measures fluid intelligence but also visual-spatial skills (Lera-Miguel et al., 2011). Ecological validity concerns the extent to which the score obtained with an instrument can be generalized to real-world settings (Dawson and Marcotte, 2017). An individual who performs at or above the normative level on a test may still experience difficulties in everyday life when requiring the cognitive function targeted by that test. For one reason, the demands placed on cognitive functions in real-world settings are more complex than in experimental settings. For another, neuropsychological tests are designed to detect clinically significant impairments in cognitive functions. This is relevant, as cognitive impairments in violent individuals are often subclinical (Ogilvie et al., 2011). Fifth, cognitive ToM was assessed verbally. Given that poverty of speech is a prominent symptom of psychotic disorders, this may have introduced bias (Sarfati et al., 1999). However, a meta-analysis of 29 studies found that individuals with schizophrenia (*N* = 1 518) performed similarly on verbal (*d* = 1.2, 95% CI 1.0–1.5) and nonverbal (*d* = 1.3, 95% CI 1.0–1.5) tests of cognitive ToM (Sprong et al., 2007). Finally, the CPT-HQ measures only one of three types of inhibition, namely selective attention. The other types are cognitive inhibition and self-control. Selective attention allows one to focus on a particular stimulus, while ignoring others. Cognitive inhibition involves the suppression of irrelevant thoughts, typically to support working memory. Self-control refers to goal-oriented regulation of thoughts and emotions (Diamond, 2013). As such, self-control may be expected to be most directly related to violent behavior. However, selective attention and self-control are highly correlated (Friedman and Miyake, 2004) and recruit largely the same neural systems (Cohen et al., 2006).

The findings of this study provide little justification for using EFs and ToM as targets for risk assessment and interventions. While most risk assessment tools do not contain items for cognitive functions (Singh et al., 2011), the small effect sizes suggest that additional items for fluid intelligence, planning and cognitive ToM would confer marginal improvements at most. For the same reason, interventions aimed at improving fluid intelligence, planning and cognitive ToM are unlikely to prevent violent behavior on their own. To improve understanding of causal mechanisms, future studies should use prospective designs and test for additional confounders and mediators.

In conclusion, we have found significant but small associations between poorer neuropsychological test performance and violent behavior in psychotic disorders for three cognitive functions: fluid intelligence, planning and cognitive ToM. This provides some empirical support for cognitive models of violent behavior. At the same time, the findings suggest that impairments in EFs and ToM have little to no value for risk assessment and interventions.

## Contributors

GROUP investigators coordinated recruitment and data collection; JL and SF designed the study; JL carried out the literature review and analyses under supervision of SF and WC; JL drafted the manuscript, and SF, WC and GROUP investigators critically reviewed and revised it. All have approved the final manuscript.

## Funding

The GROUP project was supported by the Geestkracht program of The Netherlands Organization for Health Research and Development (grant number 10-000-1001) and matching funds from the coordinating university medical centers (i.e. Academic Medical Center, Maastricht University Medical Center+, University Medical Center Groningen and University Medical Center Utrecht), their affiliated mental health care institutions (i.e. Altrecht, Arkin, Delta, Dimence, Dijk en Duin, Erasmus University Medical Center, GGNet, GGZ Breburg, GGZ Centraal, GGZ Drenthe, GGZ Eindhoven en De Kempen, GGZ Friesland, GGZ inGeest, Mondriaan, GGZ Noord-Holland-Noord, GGZ Oost-Brabant, GGZ Overpelt, GGZ Rivierduinen, Lentis, Mediant GGZ, Met GGZ, Parnassia Psycho-Medical Center, Psychiatric Center Ziekeren, Psychiatric Hospital Sancta Maria, Public Center for Mental Health Rekem, The Collaborative Antwerp Psychiatric Research Institute, Vincent van Gogh voor Geestelijke Gezondheid, Virenze riagg, University Psychiatric Center Sint Jozef, Yulius, and Zuyderland GGZ) and participating pharmaceutical companies (i.e. Lundbeck, AstraZeneca, Eli Lilly and Janssen Cilag). JL received funding from the Prins Bernhard Cultuurfonds and Dr. Hendrik Muller Fonds. SF was funded by a Wellcome Trust Senior Research Fellowship in Clinical Science (202836/Z/16/Z). The funders had no role in study design, data collection and analysis, decision to publish or preparation of the manuscript.

## Declaration of competing interest

The authors declare no conflicts of interest.

## Appendix A Supplementary data

Supplementary material Neuropsychological test procedure at baseline and results of sensitivity analyses Image 1
