# Supplementary material for: Cognition and violent behavior in psychotic disorders: A nationwide case-control study
Source: Schizophr Res Cogn. 2019 Oct 31;19:100166. doi: 10.1016/j.scog.2019.100166 (PMC6890945; doi:10.1016/j.scog.2019.100166)
Supplement: Supplementary file 1 — Supplementary material Neuropsychological test procedure at baseline and results of sensitivity analyses [file mmc1.docx]

**SUPPLEMENT**

**GROUP investigators**

Behrooz Z. Alizadeh^a^, Agna A. Bartels-Velthuis^a^, Nico J. van Beveren^b,c,d^, Richard Bruggeman^a^, Lieuwe de Haan^e^, Philippe Delespaul^f^, Carin J. Meijer^e^, Inez Myin-Germeys^g^, René S. Kahn^h^, Frederike Schirmbeck^e^, Claudia J. Simons^f,i^, Neeltje E. van Haren^h^, Jim van Os^f,j^, Ruud van Winkel^f,g^

^a^ University Psychiatry Center, University Medical Center Groningen, Groningen, the Netherlands

^b^ Antes Center for Mental Health Care, Rotterdam, the Netherlands

^c^ Department of Psychiatry, Erasmus University Medical Center, Rotterdam, the Netherlands

^d^ Department of Neuroscience, Erasmus University Medical Center, Rotterdam, the Netherlands

^e^ Department of Psychiatry, Academic Medical Center, Amsterdam, the Netherlands

^f^ Department of Psychiatry and Neuropsychology, Maastricht University Medical Center+, Maastricht, the Netherlands

^g^ Department of Neuroscience, Katholieke Universiteit Leuven, Leuven, Belgium

^h^ Department of Psychiatry, University Medical Center Utrecht, Utrecht, the Netherlands

^i^ Institute for Mental Health Care Eindhoven and De Kempen, Eindhoven, the Netherlands

^j^ Department of Psychosis Studies, King’s College London, London, UK

**Neuropsychological assessment**

The testing procedure at baseline is summarized in Table S1. Patients were assessed on all domains of the Measurement and Treatment Research to Improve Cognition in Schizophrenia consensus battery, except “visual learning and memory” (Nuechterlein et al., 2004). Testing was done in a single session and took about two hours in total. Breaks between tests were offered whenever the patient showed signs of fatigue. Below follows a description of the neuropsychological tests used in the present study.

Inhibition was measured with the Continuous Performance Test-HQ (Neuechterlein and Dawson, 1984). A total of 300 letters appear in quasi-random order on a computer screen and the subject is instructed to press the space bar on a keyboard each time the letter “Q” is preceded by the letter “H” (*k* = 28). Letters are presented for 150 ms, with intervals of 850 ms. We used three performance indicators: (i) the number of hits (i.e. correct positive responses); (ii) the mean reaction time for hits; and (iii) the number of commission errors (i.e. false positive responses).

The Response Shifting Task (RST), a modified version of the Competing Programs Task (Bilder et al., 1992), was used to assess cognitive flexibility. The RST requires set-shifting between imitation and reversal response rules. The stimulus words “left” and “right” appear quasi-randomly on a computer screen for 3 s and should be followed each time by a press on either the left (i.e. “z”) or right (i.e. “/”) side of the keyboard. In the imitation condition, the subject must press the key congruent with the meaning of the stimulus (e.g. “z” when the word “left” appears). In the reversal condition, the subject must press the key incongruent with the meaning of the stimulus (e.g. “/” when the word “left” appears). Each condition is presented twice in alternating blocks, which end after 20 responses or 8 consecutive correct responses. The subject has to rely on feedback, shown for 1 s after each response, to deduce the response rule of each block. Two performance indicators were used: (i) accuracy cost (i.e. the absolute difference between the percentage of correct responses in the imitation condition and the percentage of correct responses in the reversal condition); and (ii) reaction time cost (i.e. the absolute difference between the mean reaction time in the imitation condition and the mean reaction time in the reversal condition). The first response in each block, responses preceded by errors, responses with a reaction time shorter than 150 ms and reaction times for incorrect responses were excluded.

Fluid intelligence was measured with the Block Design subtest of the Wechsler Adult Intelligence Scale, Third Edition (Wechsler, 1997). The subject is asked to arrange 4 to 16 red and white square blocks in color patterns that match those shown on cards or made by the examiner. Each trial has a specified time limit (range: 90-240 s). Patterns that are laid incorrectly or outside the time limit are scored with 0 points. Scores for correct patterns depend on the number of tries and time to completion. If the subject lays 5 incorrect patterns in succession, the test is terminated and he or she receives a total score of 0 points. The maximum score is 68 points.

The Mazes Test of the Neuropsychological Assessment Battery (Stern and White, 2003) was used to assess planning ability. The subject has to complete 7 progressively difficult mazes with time limits (range: 30-240 s) imposed on each. An uncompleted maze is scored with 0 points. Time to completion is divided in intervals that are scored with 1 to 3 or 5 points. If the subject fails to complete 3 mazes in a row, the test is terminated and he or she receives a total score of 0 points. The maximum score is 26 points.

The Degraded Facial Affect Recognition Task (van ’t Wout et al., 2004; van Dijke et al., 2016) was used to measure affective theory of mind (ToM). Photographs of four actors (two male and two female) with angry, happy, fearful and neutral faces appear in random order on a computer screen. The subject is instructed to identify the emotion expressed in each face by pressing the key with the corresponding number (i.e. “1”-“4”). There are 16 representations of each emotion, making the total number of trials 64. To increase the contribution of top-down processes, such as attention, mental imagery and feature binding, the photographs are passed through a filter that reduces visual contrast by 30%. For the same reason, half of the trials for angry, happy and fearful faces are displayed at 75% intensity (van Dijke et al., 2016). The percentage of correctly identified emotions was used as the performance indicator.

Face recognition ability was assessed with the short form of the Benton Facial Recognition Test (BFRT) (Benton et al., 1983). The BFRT uses photographs of male and female faces with hair and clothing cropped out. The subject is asked to match a target face with 1 or 3 out of 6 test faces in 13 trials. Correctly matched faces are scored with 1 point. The maximum score is 27 points.

Cognitive ToM was measured with the Hinting Task (Corcoran et al., 1995). The Hinting Task consists of 10 short stories, each involving an interaction between two characters, that are read aloud. Each story ends with one of the characters making an implicit statement. The subject is then asked to explain what the character really meant. An immediate correct answer is scored with 2 points. In case of an incorrect response, the subject is given a hint. Subsequent responses are scored with 1 point, if correct, or 0 points, if incorrect.

**Tables**

**Table S1. Neuropsychological tests administered at baseline.**

Neuropsychological test Targeted cognitive function Reference

Word Learning Task (immediate recall)^a^ Short-term memory Brand and Jolles (1985)

Response Shifting Task^a,b^ Cognitive flexibility Bilder et al. (1992)

Continuous Performance Test-HQ^a,b^ Inhibition Neuechterlein and Dawson (1984)

WAIS-III (Digit-Symbol Coding) Processing speed Wechsler (1997)

Word Learning Task (delayed recall and recognition)^a^ Short-term memory Brand and Jolles (1985)

Degraded Facial Affect Recognition Task^a,b^ Affective ToM van ’t Wout et al. (2004)

Benton Facial Recognition Test^b^ Face recognition Benton et al. (1983)

WAIS-III (Information) Semantic memory Wechsler (1997)

WAIS-III (Arithmetic) Working memory Wechsler (1997)

WAIS-III (Block Design) Fluid intelligence Wechsler (1997)

Hinting Task Cognitive ToM Corcoran et al. (1995)

WAIS-III, Wechsler Adult Intelligence Scale, Third Edition; ToM, theory of mind.

Neuropsychological tests are listed in the order in which they were administered. Parentheses indicate subtests.

^a^ Administered using E-Prime 1.3 (Psychology Software Tools Inc., Pittsburgh).

^b^ Included in the present study.

**Table S2. Neuropsychological test performance in violent and nonviolent patients with psychotic disorders, including only patients with violence that resulted in injury.**

Unadjusted M (SD) Adjusted M (SE)^a^

Targeted cognitive function Neuropsychological test V (*n* = 56) NV (*n* = 708) V (*n* = 56 ) NV (*n* = 708) *F* (*df1*, *df2*) *p* $\text{η}_{\text{p}}^{\text{2}}$

*Executive functions*

Inhibition CPT-HQ^b^ 0.02 (0.48) 0.00 (0.55) 0.05 (0.08) 0.00 (0.02) 0.40 (1, 670) ns .001

Number of hits 26.0 (3.7) 26.4 (2.8)

Mean reaction time hits^c^ 44.1 (8.9) 42.8 (8.7)

Number of commission errors 2.1 (5.1) 3.0 (15.3)

Cognitive flexibility RST^b^ -0.17 (0.76) 0.01 (0.75) -0.20 (0.11) 0.01 (0.03) 3.69 (1, 654) ns .006

Accuracy cost 28.7 (29.5) 22.2 (23.6)

Reaction time cost^c^ 27.4 (17.2) 25.3 (18.9)

Fluid intelligence WAIS-III Block Design subtest 33.9 (17.5) 41.0 (16.9) 33.2 (2.4) 41.1 (0.6) 10.35 (1, 724) .001 .014

Planning NAB Mazes Test^d^ 15.0 (7.0) 17.0 (6.3) 14.4 (1.1) 17.0 (0.3) 5.21 (1, 424) .023 .012

*Theory of mind*

Affective DFAR 68.4 (10.3) 68.9 (10.7) 68.3 (1.5) 68.9 (0.4) 0.13 (1, 654) ns < .001

Cognitive Hinting Task 16.9 (3.5) 17.7 (2.7) 17.0 (0.4) 17.7 (0.1) 3.40 (1, 719) ns .005

M, mean; SD, standard deviation; SE, standard error; V, violent; NV, nonviolent; *df*, degrees of freedom; CPT-HQ, Continuous Performance Test-HQ; ns, nonsignificant; RST, Response Shifting Task; WAIS-III, Wechsler Adult Intelligence Scale, Third Edition; NAB, Neuropsychological Assessment Battery; DFAR, Degraded Facial Affect Recognition Task.

Due to missing data, the total number of patients varies per test.

^a^ Adjusted for age and sex.

^b^ Average of subscale *z*-scores.

^c^ In centiseconds.

^d^ Administered six years after baseline.

**Table S3. Neuropsychological test performance in violent and nonviolent patients with psychotic disorders, excluding patients with violence before illness onset.**

Unadjusted M (SD) Adjusted M (SE)^a^

Targeted cognitive function Neuropsychological test V (*n* = 130) NV (*n* = 708) V (*n* = 130) NV (*n* = 708) *F* (*df1*, *df2*) *p* $\text{η}_{\text{p}}^{\text{2}}$

*Executive functions*

Inhibition CPT-HQ^b^ 0.05 (0.50) 0.00 (0.55) 0.06 (0.05) -0.01 (0.02) 1.38 (1, 739) ns .002

Number of hits 26.2 (2.6) 26.4 (2.8)

Mean reaction time hits^c^ 44.2 (7.9) 42.8 (8.7)

Number of commission errors 2.3 (9.5) 3.0 (15.3)

Cognitive flexibility RST^b^ -0.04 (0.80) 0.01 (0.75) -0.05 (0.07) 0.01 (0.03) 0.74 (1, 719) ns .001

Accuracy cost 23.3 (23.8) 22.2 (23.6)

Reaction time cost^c^ 26.1 (18.9) 25.3 (18.9)

Fluid intelligence WAIS-III Block Design subtest 37.6 (17.5) 41.0 (16.9) 37.1 (1.5) 41.1 (0.6) 5.76 (1, 797) .017 .007

Planning NAB Mazes Test^d^ 15.3 (6.7) 17.0 (6.3) 15.0 (0.7) 17.1 (0.3) 7.32 (1, 468) .007 .015

*Theory of mind*

Affective DFAR 68.8 (9.9) 68.9 (10.7) 68.7 (1.0) 68.9 (0.4) 0.03 (1, 717) ns < .001

Cognitive Hinting Task 17.0 (2.9) 17.7 (2.7) 17.0 (0.2) 17.7 (0.1) 6.09 (1, 789) .014 .008

M, mean; SD, standard deviation; SE, standard error; V, violent; NV, nonviolent; *df*, degrees of freedom; CPT-HQ, Continuous Performance Test-HQ; ns, nonsignificant; RST, Response Shifting Task; WAIS-III, Wechsler Adult Intelligence Scale, Third Edition; NAB, Neuropsychological Assessment Battery; DFAR, Degraded Facial Affect Recognition Task.

Due to missing data, the total number of patients varies per test.

^a^ Adjusted for age and sex.

^b^ Average of subscale *z*-scores.

^c^ In centiseconds.

^d^ Administered six years after baseline.

**Table S4. Neuropsychological test performance in violent and nonviolent patients with psychotic disorders, excluding patients with PANSS total scores of 95 or higher.**

Unadjusted M (SD) Adjusted M (SE)^a^

Targeted cognitive function Neuropsychological test V (*n* = 163) NV (*n* = 631) V (*n* = 163) NV (*n* = 631) *F* (*df1*, *df2*) *p* $\text{η}_{\text{p}}^{\text{2}}$

*Executive functions*

Inhibition CPT-HQ^b^ 0.00 (0.54) 0.00 (0.56) 0.01 (0.05) -0.01 (0.02) 0.10 (1, 704) ns < .001

Number of hits 26.1 (3.2) 26.4 (2.8)

Mean reaction time hits^c^ 43.7 (8.0) 42.8 (8.7)

Number of commission errors 2.8 (11.4) 3.0 (15.4)

Cognitive flexibility RST^b^ -0.05 (0.78) 0.02 (0.74) -0.07 (0.06) 0.03 (0.03) 1.83 (1, 685) ns .003

Accuracy cost 23.1 (24.5) 21.7 (23.1)

Reaction time cost^c^ 27.1 (19.1) 25.4 (18.6)

Fluid intelligence WAIS-III Block Design subtest 38.3 (17.5) 41.5 (16.9) 37.9 (1.4) 41.6 (0.7) 5.94 (1, 766) .015 .008

Planning NAB Mazes Test^d^ 15.9 (6.6) 16.9 (6.3) 15.5 (0.6) 17.0 (0.3) 4.87 (1, 466) .028 .010

*Theory of mind*

Affective DFAR 69.5 (9.7) 69.0 (10.5) 69.4 (0.9) 69.0 (0.4) 0.11 (1, 693) ns < .001

Cognitive Hinting Task 17.0 (3.0) 17.8 (2.7) 17.0 (0.2) 17.8 (0.1) 8.85 (1, 758) .003 .012

PANSS, Positive and Negative Syndrome Scale; M, mean; SD, standard deviation; SE, standard error; V, violent; NV, nonviolent; *df*, degrees of freedom; CPT-HQ, Continuous Performance Test-HQ; ns, nonsignificant; RST, Response Shifting Task; WAIS-III, Wechsler Adult Intelligence Scale, Third Edition; NAB, Neuropsychological Assessment Battery; DFAR, Degraded Facial Affect Recognition Task.

Due to missing data, the total number of patients varies per test.

^a^ Adjusted for age and sex.

^b^ Average of subscale *z*-scores.

^c^ In centiseconds.

^d^ Administered six years after baseline.

**Table S5. Neuropsychological test performance in violent and nonviolent patients with psychotic disorders, excluding patients with substance misuse.**

Unadjusted M (SD) Adjusted M (SE)^a^

Targeted cognitive function Neuropsychological test V (*n* = 55) NV (*n* = 274) V (*n* = 55) NV (*n* = 274) *F* (*df1*, *df2*) *p* $\text{η}_{\text{p}}^{\text{2}}$

*Executive functions*

Inhibition CPT-HQ^b^ 0.05 (0.31) -0.01 (0.65) 0.06 (0.09) -0.02 (0.04) 0.70 (1, 291) ns .002

Number of hits 26.2 (2.3) 26.4 (2.7)

Mean reaction time hits^c^ 43.9 (7.5) 43.2 (8.9)

Number of commission errors 1.3 (2.0) 3.9 (20.3)

Cognitive flexibility RST^b^ 0.02 (0.72) 0.03 (0.79) 0.01 (0.11) 0.03 (0.05) 0.03 (1, 287) ns < .001

Accuracy cost 25.2 (25.7) 23.2 (24.5)

Reaction time cost^c^ 21.9 (15.0) 24.1 (19.2)

Fluid intelligence WAIS-III Block Design subtest 35.3 (16.2) 40.6 (17.0) 34.9 (2.3) 40.7 (1.0) 5.47 (1, 317) .020 .017

Planning NAB Mazes Test^d^ 14.0 (6.7) 16.6 (6.4) 13.7 (1.1) 16.6 (0.5) 6.20 (1, 190) .014 .032

*Theory of mind*

Affective DFAR 68.4 (12.0) 68.2 (10.9) 68.4 (1.6) 68.2 (0.7) 0.03 (1, 282) ns < .001

Cognitive Hinting Task 16.6 (3.0) 17.7 (2.7) 16.6 (0.4) 17.6 (0.2) 6.03 (1, 313) .015 .019

M, mean; SD, standard deviation; SE, standard error; V, violent; NV, nonviolent; *df*, degrees of freedom; CPT-HQ, Continuous Performance Test-HQ; ns, nonsignificant; RST, Response Shifting Task; WAIS-III, Wechsler Adult Intelligence Scale, Third Edition; NAB, Neuropsychological Assessment Battery; DFAR, Degraded Facial Affect Recognition Task.

Due to missing data, the total number of patients varies per test.

^a^ Adjusted for age and sex.

^b^ Average of subscale *z*-scores.

^c^ In centiseconds.

^d^ Administered six years after baseline.

**Table S6. Neuropsychological test performance in violent and nonviolent patients with psychotic disorders, including only patients with a secondary education.**

Unadjusted M (SD) Adjusted M (SE)^a^

Targeted cognitive function Neuropsychological test V (*n* = 146) NV (*n* = 620) V (*n* = 146) NV (*n* = 620) *F* (*df1*, *df2*) *p* $\text{η}_{\text{p}}^{\text{2}}$

*Executive functions*

Inhibition CPT-HQ^b^ 0.02 (0.51) 0.03 (0.48) 0.03 (0.04) 0.03 (0.02) < 0.01 (1, 674) ns < .001

Number of hits 26.2 (2.6) 26.5 (2.6)

Mean reaction time hits^c^ 43.8 (7.9) 42.9 (8.7)

Number of commission errors 2.7 (11.7) 2.4 (11.1)

Cognitive flexibility RST^b^ 0.02 (0.70) 0.00 (0.76) 0.00 (0.07) 0.00 (0.03) < 0.01 (1, 657) ns < .001

Accuracy cost 21.3 (22.4) 21.7 (23.4)

Reaction time cost^c^ 25.6 (18.2) 26.1 (19.3)

Fluid intelligence WAIS-III Block Design subtest 39.8 (17.7) 42.3 (16.4) 39.4 (1.4) 42.3 (0.7) 3.44 (1, 727) ns .005

Planning NAB Mazes Test^d^ 16.2 (6.4) 17.1 (6.2) 15.8 (0.6) 17.2 (0.3) 4.14 (1, 473) .042 .009

*Theory of mind*

Affective DFAR 69.4 (10.4) 68.8 (10.4) 69.2 (0.9) 68.9 (0.4) 0.14 (1, 658) ns < .001

Cognitive Hinting Task 17.0 (2.9) 17.9 (2.6) 17.0 (0.2) 17.9 (0.1) 12.53 (1, 720) < .001 .017

M, mean; SD, standard deviation; SE, standard error; V, violent; NV, nonviolent; *df*, degrees of freedom; CPT-HQ, Continuous Performance Test-HQ; ns, nonsignificant; RST, Response Shifting Task; WAIS-III, Wechsler Adult Intelligence Scale, Third Edition; NAB, Neuropsychological Assessment Battery; DFAR, Degraded Facial Affect Recognition Task.

Due to missing data, the total number of patients varies per test.

^a^ Adjusted for age and sex.

^b^ Average of subscale *z*-scores.

^c^ In centiseconds.

^d^ Administered six years after baseline.

**References**

Benton, A.L., Sivan, A.B., Hamsher, K.S., Varney, N.R., Spreen, O., 1983. Benton’s Test of Facial Recognition. Oxford University Press, New York.

Bilder, R.M., Turkel, E., Lipschutz-Broch, L., Lieberman, J.A., 1992. Antipsychotic medication effects on neuropsychological functions. Psychopharmacol. Bull. 28 (4), 353-366.

Brand N., Jolles J., 1985. Learning and retrieval rate of words presented auditorily and visually. J. Gen. Psychol. 112 (2), 201-210.

Corcoran, R., Mercer, G., Frith, C.D., 1995. Schizophrenia, symptomatology and social inference: Investigating “theory of mind” in people with schizophrenia. Schizophr. Res. 17 (1), 5-13.

Neuechterlein, K.H., Dawson, M.E., 1984. Information processing and attentional functioning in the developmental course of schizophrenic disorders. Schizophr. Bull. 10 (2), 160-203.

Nuechterlein, K.H., Barch, D.M., Gold, J.M., Goldberg, T.E., Green, M.F., Heaton, R.K., 2004. Identification of separable cognitive factors in schizophrenia. Schizophr. Res. 72 (1), 29-39.

Stern, R.A., White, T., 2003. Neuropsychological Assessment Battery. Psychological Assessment Resources, Lutz.

van Dijke, A., van ’t Wout, M., Ford, J.D., Aleman, A., 2016. Deficits in degraded facial affect labeling in schizophrenia and borderline personality disorder. PLoS One. 11 (6), e0154145.

van ’t Wout, M., Aleman, A., Kessels, R.P., Laroi, F., Kahn, R.S., 2004. Emotional processing in a non-clinical psychosis-prone sample. Schizophr. Res. 68 (2-3), 271-281.

Wechsler, D., 1997. Wechsler Adult Intelligence Scale, WAIS-III. Psychological Corporation, San Antonio.
